# Lower glycemia levels in subjects with excessive erythrocytosis during the oral glucose tolerance test living in conditions of severe hypoxia

**DOI:** 10.3389/fphys.2024.1387132

**Published:** 2024-04-09

**Authors:** Kely Melina Vilca Coaquira, Rossela Alejandra Rojas Chambilla, Jeancarlo Tejada Flores, Henry Oscar Tintaya Ramos, Mariela Mercedes Quispe Trujillo, Solanyela Anny Quispe Humpiri, Ángel Gabriel Calisaya Huacasi, Yony M. Pino Vanegas, Gilberto Félix Peña Vicuña, Alberto Alcibiades Salazar Granara, Ana Lucia Tacuna Calderon, Nancy Monica Garcia Bedoya, Moua Yang, Ivan Hancco Zirena

**Affiliations:** ^1^ Facultad de Medicina Humana, Universidad Nacional Del Altiplano, Puno, Perú; ^2^ ACEM UNA, Puno, Perú; ^3^ Facultad De Educación, Escuela Profesional De Educación Física, UNA Puno, Puno, Perú; ^4^ Instituto De Investigación en Medicina De Altura (CIMA), Facultad De Medicina Humana, Universidad De San Martín De Porres, Lima, Perú; ^5^ Facultad De Ciencias De La Educación, Escuela Profesional De Educación Inicial, UNA Puno, Puno, Perú; ^6^ Division of Hemostasis and Thrombosis, Beth Israel Deaconess Medical Center and Harvard Medical School, Boston, MA, United States

**Keywords:** glucose, excessive erythrocytosis, hypoxia, glucose tolerance test, high altitude

## Abstract

**Background:**

Previous studies showed that residents of higher elevations have lower glucose levels. Our objective in this study is to determine the basal and postprandial glucose levels in apparently healthy permanent residents of the miner population center of La Rinconada located 5100 meters (m) above sea level.

**Method:**

Forty male permanent residents of the Rinconada miner population center were studied. The oral glucose tolerance test was used to evaluate basal and postprandial glycemia levels at 1, 2, and 3 h.

**Results:**

The individuals had a mean age of 43.95 ± 8.54 years. Basal glycemia in subjects without excessive erythrocytosis (EE) was 73.3 ± 7.9 mg/dL, while levels in patients with EE were 57.98 ± 7.38 mg/dL. In the postprandial period, at 1 h after oral glucose overload, a mean value of 76.35 ± 13.53 mg/dL was observed in subjects with EE compared to 94.68 ± 9.98 mg/dL in subjects without EE. After 2 h, subjects with EE had a glycemia level of 72.91 ± 9.17 mg/dL EE compared to 90.73 ± 13.86 mg/dL without EE. At 3 h, the average glycemia level in subjects with EE was 70.77 ± 8.73 mg/dL compared to 87.79 ± 14.16 mg/dL in those without EE.

**Conclusion:**

These findings suggest that under hypoxic conditions, glycemia levels are lower in both subjects with and without EE, having obtained lower levels in subjects with EE in relation to those with normal values of Hb and Hct. The results of this study indicate that in the conditions of severe hypoxia, blood glucose levels are below the values considered normal for sea level.

## 1 Introduction

The inhabitants of high-altitude regions experience a unique environment with characteristics including dryness, intense solar radiation, cold, and hypoxia, leading to profound physiological changes. At higher altitudes, barometric pressure decreases, which produces a decrease in inspired air (Hurtado, 1964; Beall, 2007; Paralikar and Paralikar, 2010; Dhar, et al., 2014; Tymko, et al., 2017).

At the level of the endocrine system, physiologic adaptation aims to regulate metabolism and ensure optimal energy production for cellular function (Gonzales, 2001; Gonzales, et al., 2011). Oxygen deprivation triggers adjustments to enhance energy production and prevent cellular damage. High-altitude residents exhibit lower basal glucose and insulin levels, likely due to a preference for metabolizing glucose as the main carbon source (Oltmanns et al., 2004; Thomas et al., 2010; Van Hulten et al., 2021). The lower prevalence of obesity and diabetes in these populations is attributed to increased energy production to circumvent altitude challenges, including the challenge of reduced oxygen availability. This increased energy demand promotes substrate consumption, minimizes adipose tissue formation, and fosters energy homeostasis for cellular function. Over time, efficient adaptation has evolved to counteract the adverse environmental effects of high altitude (Hochachka et al., 1996; Woolcott et al., 2014; Horscroft et al., 2017; West, 2017).

The oral glucose tolerance test is a cost-effective and straightforward procedure, assessing how the body processes glucose from the blood to tissues (Eyth et al., 2023). Leveraging this assay, our study focused on seemingly healthy individuals residing permanently at 5100 m above sea level (m.a.s.l.) in a severe hypoxic environment. Our goal was to determine basal and postprandial glycemia levels and examine the potential impact of hypoxia on glucose metabolism. We aimed to evaluate whether these conditions could serve as a protective factor against metabolic diseases, including diabetes mellitus and obesity.

## 2 Materials and methods

### 2.1 Study population

This study involved 40 male subjects residing for more than a year at the La Rinconada Miner Population Center, which is situated over 5100 m.a.s.l. Exclusion criteria included acute or chronic illnesses, harmful habits (e.g*.*, alcohol, smoking, and coca consumption), regular medication use, or any condition affecting glycemic levels.

### 2.2 Procedure

Information on age, sex, weight, height, length of residence in La Rinconada, and the place of origin was obtained during a medical consultation organized by the miners’ association of La Rinconada.

Vital signs, including systolic and diastolic blood pressure (SBP and DBP) and heart rate (HR), were recorded using a digital sphygmomanometer (Riester brand ri-champion adult digital arm sphygmomanometer, measuring range 30–280 mmHg and heart rate 40 to 200 beats per minute). A pulse oximeter (Nellcor^®^ OxiMax^®^ N-65 hand-held pulse oximeter, Digicare Biomedical brand, 1% saturation resolution and heart rate range 30–235 beats per minute) was used to measure oxygen saturation (SatO_2_). In addition, a Camry model EB9068-59 digital scale and a measuring rod were used to estimate the weight and height of the subjects evaluated, respectively, and for subsequent calculation of the body mass index (BMI).

For blood sampling, aseptic measures were taken using an alcohol-moistened cotton swab on the patient’s middle or ring fingers, followed by capillary puncture using a sterile lancet. After removing the lancet, a waiting period allowed for the spontaneous formation of a blood drop. The initial two drops were removed with a cotton swab to prevent errors, ensuring the third drop was of sufficient volume to fill the microcuvette. Afterward, the area was cleaned with a dry cotton swab, and the second and third samples were collected for the microcentrifuge tube and glucometer test strip, respectively. Finally, the area was disinfected, and an adhesive bandage was applied.

Hemoglobin levels were assessed using a HemoCue HB 201+ portable hemoglobinometer, using the azide-methemoglobin method within a measurement range of 0–25.6 g/dL. The microcuvette with the blood sample was positioned for analysis. Hematocrit measurements were conducted using a HemataStat II microcentrifuge from EKF Diagnostics, which involves the placement of the tube for centrifugation and subsequent measurements. Blood glucose levels were determined using a portable Accu-Check Active IV glucometer by placing the test strip with the blood drop.

Postprandial glycemia values were obtained through an oral glucose tolerance test. After obtaining basal glycemia values, patients ingested a liquid containing 75 g of glucose. Subsequent measurements were taken at the first, second, and third hours after the ingestion to determine postprandial glycemia values.

The current international consensus on chronic mountain sickness (León-Velarde, et al., 2005) was used to consider excessive erythrocytosis (EE).

### 2.3 Statistical analysis

The mean with its respective standard deviation (SD) of the variables was established for subjects with and without EE. Normality was determined using the Shapiro–Wilk and Kolmogorov–Smirnov tests, resulting in all our variables being normally distributed. To establish the relationship between two variables, Student’s t-test was used. Statistical processing was carried out using the IBM SPSS version 25 statistical package.

### 2.4 Ethical aspects

Before starting the measurements, each procedure and the usefulness of the results were explained in detail so that the subjects could sign the informed consent, authorizing the respective measurements.

This study was approved by the USMP ethics committee with the International Registry Federalwide Assurance (FWA) for the Protection of Human Subjects for International (No. 00015320, IRB No. 00003251).

## 3 Results

The goal of this study was to assess basal and postprandial glycemia levels in subjects with and without EE utilizing the glucose tolerance test, a cost-effective and straightforward method. Two groups were selected: subjects with EE and without EE. Notably, this is the first study to consider Hb, Hct, DBP, SBP, and oxygen saturation (SatO_2_) values, which are important parameters that may vary with altitude. These parameters and their potential correlation with glucose levels remain uncertain.

Both groups had similar ages, as indicated in Table 1. SatO_2_ levels were comparable between the two groups, while the body mass index (BMI) showed a statistically significant but slightly elevated level in patients with EE.

**TABLE 1:** Main characteristics of the subjects studied.

	Without excessive erythrocytosis Hb < 21	With excessive erythrocytosis Hb ≥ 21	*t*-Test
	Mean ± SD n = 22	Mean ± SD n = 18	P (<0.05)
**Age (Years)**	43.2 ± 10.2	44.5 ± 7.0	0.659
**Heart rate (HR) beats per minute (bpm)**	75.0 ± 12.6	81.3 ± 15.1	0.166
**Oxygen saturation (SatO**_**2**_ **) (%)**	80.1 ± 3.5	78.4 ± 4.1	0.177
**Body mass index (BMI) (Kg/m** ^**2**^ **)**	25.0 ± 2.7	26.8 ± 1.7	0.019
**Hemoglobin (Hb) (g/dL)**	18.9 ± 1.1	23.9 ± 1.7	<0.001
**Hematocrit (Hct) (%)**	59.7 ± 6.4	75.3 ± 5.6	<0.001
**Systolic blood pressure (SBP) (mmHg)**	115.0 ± 15.5	120.0 ± 14.8	0.311
**Diastolic blood pressure (DBP) (mmHg)**	72.4 ± 8.7	79.4 ± 11.5	0.040
**Basal blood glucose (mg/dL)**	73.3 ± 7.9	58.6 ± 7.6	<0.001

**FIGURE 1:**
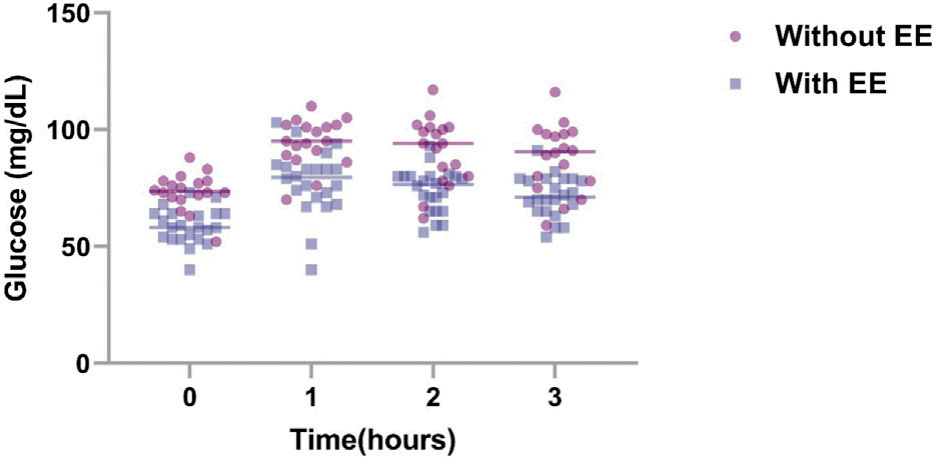
Glycemia values over time in subjects with and without EE. To determine the basal and postprandial glycemia levels in individuals who live at 5000 m, individuals with EE (n = 22) and without EE (n = 18) were subjected to the oral glucose tolerance test. Blood glucose was measured every hour up to 3 h, and the data were presented as mean +SD. EE, excessive erythrocytosis; SD, standard deviation.

**FIGURE 2:**
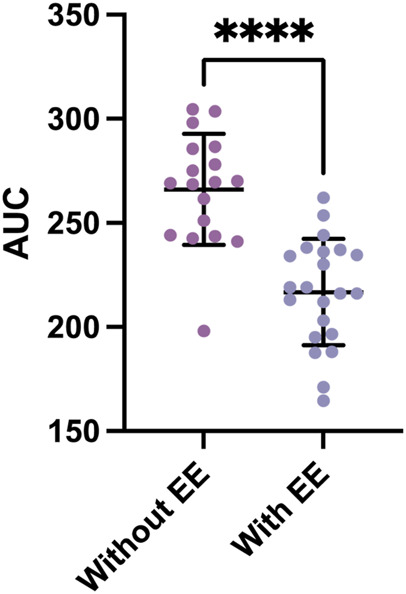
Area under the curve for the glucose tolerance test between subjects with and without EE. The areas under the curve (AUC) from Figure 1 were graphed (without EE, N = 22; without EE, N = 18). Data represented as mean + SD. **** = *p* < 0.0001.

**FIGURE 3:**
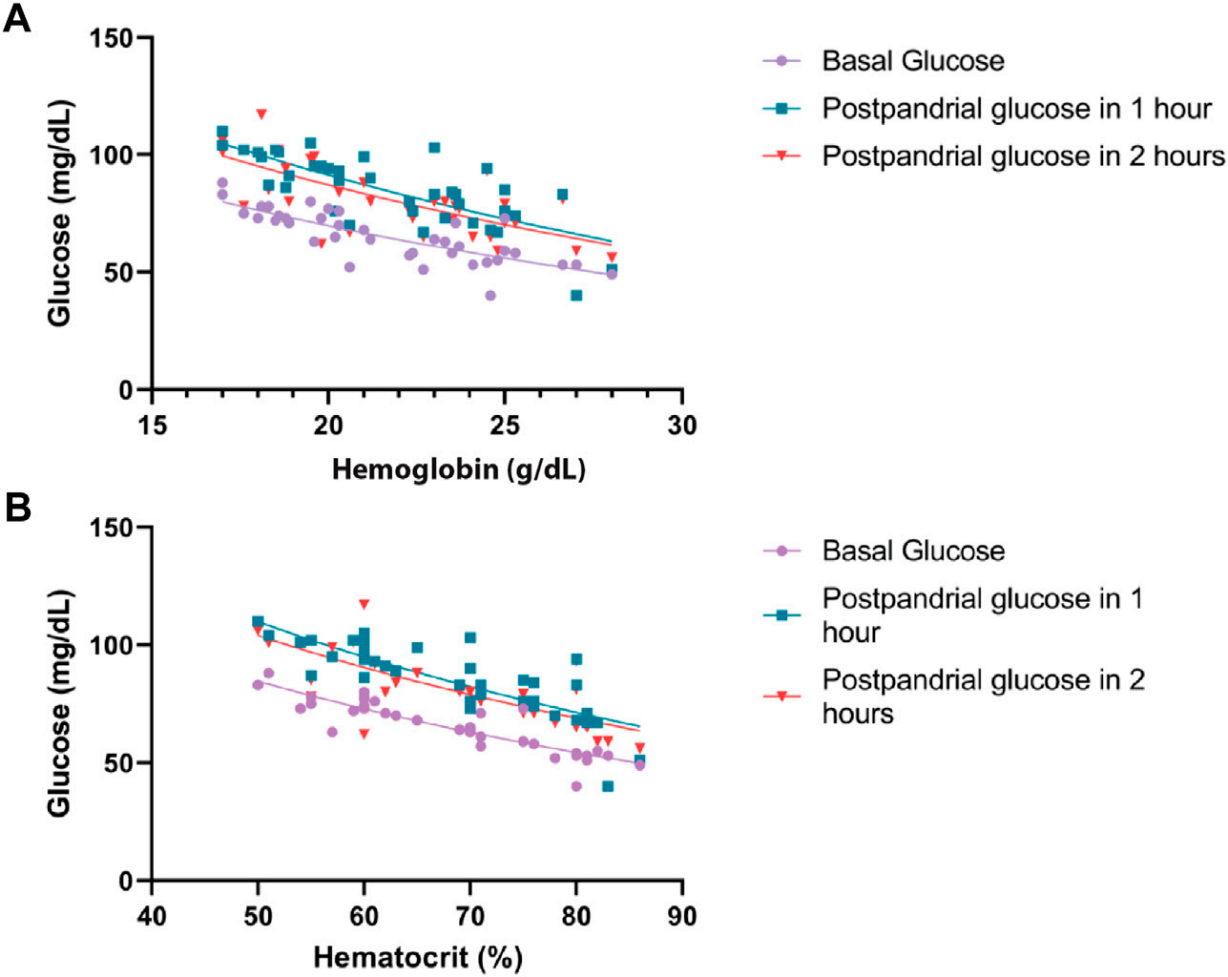
Relationship between glucose with hemoglobin and hematocrit at basal, 1 h, and 2 h postprandial. **(A)** Correlation between hemoglobin and glucose levels. *p* < 0.001. **(B)** Correlation between hematocrit and glucose levels. *p* < 0.001.

Among the vital signs assessed, subjects with EE exhibited slightly higher HR, likely compensating for the increased erythrocyte mass necessitating greater cardiac output. SatO_2_ was marginally lower in individuals with EE, which is consistent with the lower barometric pressure in the region that contributes to hypoxemia and elevated red blood cell count. Subjects with EE demonstrated a higher BMI than those without EE (*p* = 0.019), which is consistent with previous studies, linking insulin resistance to weight gain. Notably, glycemia values in both groups were within normal ranges. This defies the expectations of hyperglycemia in higher-altitude residents. The criteria for defining EE in this study adhere to the CMM consensus (León-Velarde, et al., 2005). Although SBP was higher in EE subjects, it was not significantly different between groups (*p* = 0.311) with values within the normal range of <140/90 mmHg (Zhou, et al., 2021). DBP was also higher in EE subjects (*p* = 0.040), showing a substantial difference in both groups, and is consistent with previous findings (Corante, et al., 2018). During the oral glucose tolerance test, basal and postprandial values in both groups were lower than reference values for the test (Figure 1; *p* < 0.001). Subjects with EE showed lower glycemia levels compared to subjects without EE during the test (Figure 2; *p* < 0.0001). Glycemia levels were also compared with the levels of Hb and Hct. An inverse correlation was found between Hb and Hct values and glycemia (Figures 3A, B).

## 4 Discussion

The age of the subjects studied in both groups was similar in order to avoid factors that contribute to variance based on age, sex, and other associated factors. One of the most important parameters taken into account was the period of residence. Permanent residents were defined as those who lived for more than 1 year and stayed for a period of less than 3 days per month in areas of lower altitude since most of the inhabitants work 5 days a week. After this period, the subjects descend to their villages of origin, which is located in areas of lower altitude. During periods of long holidays and vacation, they reside longer in lower altitudes, which is a condition that prevents adequate adaptation. To avoid these confounding factors, we have selected individuals who have this characteristic. Another important condition that has been taken into account is their health status (e.g., presence of diseases) that may alter the parameters obtained. In this study, only apparently healthy subjects have been evaluated without symptoms associated with any disease.

This study provides a preliminary exploration of metabolic characteristics in a population adapted to a severe hypoxic environment while sustaining healthy living conditions. Importantly, the subjects are seemingly healthy youthful individuals and were engaged in physically demanding mining activities. This may explain the significant energy expenditure and metabolic demands necessary to compensate for this environment. The evidence for this was provided by normal ranges of vital signs (e.g., blood pressure and heart rate), while Hb and Hct values align with expectations for high altitude. These factors collectively define the group as free from chronic diseases.

The greater erythrocyte cell mass may increase glucose consumption, leading to reduced basal and postprandial glycemia in subjects with EE. This discrepancy, approximately 10 mg/dL when comparing both groups, is noteworthy. A potential mechanism accounting for lower blood glucose values than those in sea-level subjects involves the activation of anaerobic metabolism (Trayhurn, 2013; Görgens et al., 2017; Lempesis et al., 2020), which requires further investigation. In the studies carried out in the Himalayas (Okumiya K et al., 2010), an important association has been observed between Hb and Hct values and hyperglycemia. It was observed that the higher Hb and Hct levels were inversely correlated with lower glycemia values. This difference could be due to older, diabetic, and hypertensive subjects evaluated. In our study, the majority of the subjects were young and apparently healthy, and the differences in both groups were the Hb and Hct values. Importantly, diabetes is lower at high-altitude regions (4.5%) compared to cities that are at sea level (8.2%), while impaired fasting glucose has a prevalence of 26.4% at sea level relative to 17.4% in high-altitude regions (Seclen et al., 2015). This information coincides with reports presented by the Peruvian Minister of Health (National Center for Epidemiology, prevention and control of P diseases, 2022). It is important to highlight that in studies carried out at different altitudes, such as in Huancayo located at 3200 m and Cerro de Pasco at 4300 m, blood glucose levels of 52.7 mg/dL and 67 mg/dL were found, respectively (Castillo Sayan, 2015, Villena JE, 2001). In these studies, the average basal glycemia level in Lima, a city at sea level, was at 73 mg/dL (Castillo Sayan, 2015, Villena JE, 2001). These findings suggest lower glycemic values at high altitude; however, glycemic values in relation to Hb and Hct have not been evaluated.

An additional significant finding is the elevated basal and postprandial glycemic values concerning normal BMI and obese subjects relative to the values observed in individuals residing at sea level (Hocking et al., 2013; Villena Chávez, 1998). The levels in this study remain lower and did not surpass 100 mg/dL, which consistently falls under the threshold considered normal in the oral glucose tolerance test. It is noteworthy that the minimal differences observed in basal glycemia between the two groups (normal BMI and overweight) became more pronounced at 2 and 3 hours. In addition, the difference in BMI was minimal, and no patient had obesity. In previous studies carried out in Tibet, an important relationship was observed among ferritin levels, high levels of Hb and Hct, and environmental factors that make the prevalence of diabetes higher in this population (Okumiya K et al., 2008; Okumiya K et al., 2010; Okumiya K et al., 2016; Okumiya K et al., 2022). In the present study, we have not evaluated these variables and their association with glycemia levels. In addition, it is important to mention that the study site is above 5000 m of altitude, while the previous studies have been carried out below 4300 m (Okumiya K et al., 2008; Okumiya K et al., 2010). Nonetheless, our findings coincide with similar studies revealing lower blood glucose levels in permanent high-altitude residents (Lindgärde et al., 2004; Woolcott et al., 2014).

In addition to the previously described mechanisms, the observed phenomenon may be attributed to an elevated adenosine monophosphate to adenosine triphosphate (AMP/ATP) ratio. This ratio enhances insulin-independent glucose uptake (Broocks et al., 1991; Siques et al., 2018) and occurs particularly in individuals engaging in physical activity under hypoxic conditions. The metabolic shift to the elevated AMP/ATP ratio could be present in the subjects in this study. This effect is associated with increased cortisol concentration through the activation of CRF receptor type 1 (CRFR1) (Singh et al., 1996; Vats et al., 1999; Chen et al., 2007a). Furthermore, the greater glucose consumption due to an increased erythrocyte mass contributes to lower glucose levels in subjects with EE.

Our findings align with studies indicating lower prevalence of obesity and diabetes within a population at higher altitude compared to sea-level region, likely from enduring hypoxic conditions (Lindgärde et al., 2004; Woolcott et al., 2014). This suggests that prolonged residence at a high altitude could serve as a protective factor against metabolic diseases. However, instrument precision in measuring blood glucose concentration in the presence of high hematocrit is essential. Discrepancies in food consumption between population may also contribute to these variations (Tschop and Morrison, 2001; Chen et al., 2007b; De Theije et al., 2013; Thomas et al., 2017).

Animal models of hypoxia reinforce the findings in this study. Mice that consume similar levels of food display lower glucose values under hypoxia (Dhar et al., 2014; Vogel et al., 2018). This corroborates the impact of hypoxia on blood glucose levels. Moreover, alterations in insulin sensitivity to glucose may promote its utilization as a carbon source over lipid oxidation (Goossens et al., 2011; Goossens et al., 2018). The adaptation potentially lowers basal glucose levels, reduces oxygen consumption, and minimizes the production of reactive oxygen byproducts (Sparks, 2017; West, 2017).

In conclusion**,** the hypoxic environment poses significant challenges, yet it might have adaptive values to protect against metabolic disorders. Based on our findings, designing a study in patients or conducting experiments with animal models of metabolic disorders in a hypoxic environment would be valuable. Such findings could potentially identify mechanistic nodes of hypoxia in managing glycemia. Moreover, these studies might identify therapeutic avenues for addressing other metabolic disorders including dyslipidemia, chronic inflammation, and aging.

## 5 Limitations

A limitation in the present study was the method used to measure blood glucose levels. In the present study, we used a hemoglucotest that was previously calibrated against other laboratory methods. Although the hemoglucotest showed similar results during calibration, it would be necessary to validate the findings with a more precise method of measuring blood glucose levels. Our retrospective measurements were part of a larger study. As such, obtaining further samples for other variables, including measurements at sea level in the same subjects, is a major limitation. Along this line, our study is inclusive of healthy individuals only with EE as the only apparent variable at the time of subject enrollment. Further characterization of individuals with metabolic disorders, high-altitude-related symptoms, and sleep habits would be informative. It would also be essential to carry out a broad epidemiological and prospective study in order to understand the behavior of hyperglycemia and diabetes in high-altitude regions. Such studies could be designed to account for the differences in location. It would also be necessary to conduct studies on diabetic patients who migrate from low-lying areas to high-altitude regions to further correlate glycemia levels with altitude changes.

## Data Availability

The original contributions presented in the study are included in the article/Supplementary Material; further inquiries can be directed to the corresponding authors.
